# Synthesis and Properties of a Chiroptically Active Oligomer from 3,4-Ethylenedioxythiophene and (–)-Myrtenal

**DOI:** 10.3390/ma4061013

**Published:** 2011-05-30

**Authors:** Hirotsugu Kawashima, Hiromasa Goto

**Affiliations:** Institute of Materials Science, Graduate School of Pure and Applied Sciences, University of Tsukuba, Tsukuba, Ibaraki 305-8573, Japan; E-Mail: s-hkawashima@ims.tsukuba.ac.jp

**Keywords:** poly(3,4-ethylenedioxythiophene), chiroptically active materials, π-conjugated materials

## Abstract

Oxidative polycondensation of 3,4-ethylenedioxythiophene and (–)-myrtenal was carried out with POCl_3_. A π-conjugated system thus constructed consists of aromatic and quinoidal alternating structure linked via methine groups. We examined iodine doping effect for the resultant material with electron spin resonance spectroscopy. Circular dichroism spectra in chloroform solution showed blue-shift with increase of iodine concentration. This result indicates that the doping process can tune chiroptical activity of the chiral π-conjugated system.

## 1. Introduction

**Scheme 1:**
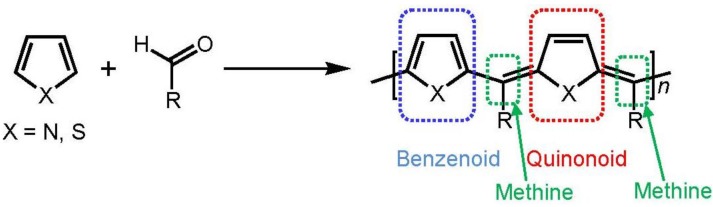
Dehydrative polycondensation for methine type polymer.

**Scheme 2:**
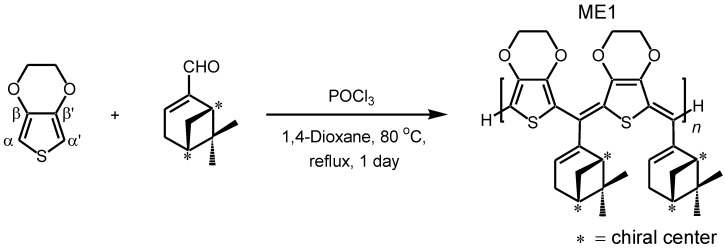
Synthesis of methine bridged oligomer as a main product from 3,4-ethylenedioxythiophene (EDOT) and (–)-myrtenal.

Many kinds of conjugated polymers have been widely studied for applications, such as transparent conductors, light-emitting diodes, thin film transistors, and photovoltaic devices [1,2,3,4,5,6,7,8,9]. Low-bandgap conjugated polymer is of interest because of its sensitivity in optical and electrical impulsions for external stimuli [10,11,12,13,14]. Poly(3,4-ethylenedioxythiophene) (PEDOT) and its derivatives are widely studied conjugated polymers due to their relatively low ionization potential, high electrical conductivity, and good stability [15,16,17,18,19,20]. A conjugated polymer which has benzenoid and quinonoid alternating structure in the main chain bridged via methine group (methine polymer) has been paid much attention because of the relatively small-bandgap [21,22,23,24,25,26,27]. Polycondensation between arylene unit and aldehyde group in the presence of sulfuric acid has been developed [28,29]. The polymerization is shown in Scheme 1. We synthesized a methine oligomer (abbreviated as ME1) from EDOT and (–)-myrtenal, a chiral compound (Scheme 2). Chiroptical activity and doping effect for the resultant thus prepared is examined based on consideration of high sensitivity derived from low-bandgap.

## 2. Experimental Section

Polycondensation between aldehydes and thiophenes provides Novolac-type polymers [30]. In this reaction, the protonated aldehyde electrophile can sequentially react with active site (α,α'-position) of thiophene. Chen and Jenekhe proved following elimination of protons at methine linkages by sulfuric acid for producing full π-conjugated skeleton [29].

Polycondensation of pyrrole and 1-dodecanal was carried out for obtaining polypyrrole-methine type π-conjugated polymers [31]. The Rothemund type reaction for synthesis of porphyrine can be applied for polycondensation between EDOT and chiral aldehydes for construction of π-conjugated main chain [32,33]. In this case, bulky group in the substituents can prevent a formation of cyclic compounds.

Chemicals were purchased from Tokyo Kasei and Aldrich and used as received. ^1^H NMR measurements of (–)-myrtenal and ME1 were performed in CDCl_3_ with ECS 400 spectrometer (JEOL) at room temperature. Chemical shifts are reported in ppm downfield from SiMe_4_, using the solvent’s residual signal as an internal reference. Fourier transfer infrared (FTIR) absorption spectra of ME1 and (–)-myrtenal were obtained by FT/IR-300 spectrometer (Jasco), with the KBr method. Molecular weights of ME1 were determined by gel permeation chromatography (GPC) with MIXED-D HPLC column (Polymer Laboratories), PU-980 HPLC pump (Jasco) and MD-915 multiwavelength detector (Jasco), with THF used as the solvent, with the instruments calibrated by polystyrene standard. UV-visible absorption spectra were recorded on V-630 UV-vis optical absorption spectrometer (Jasco). Circular dichroism (CD) spectra were obtained with J-720 spectrometer (Jasco). Electron spin resonance (ESR) spectra were taken at room temperature using JES-TE200 ESR spectrometer (JEOL) during *in-situ* vapor phase doping process with iodine.

The target material was synthesized by dehydrative polycondensation between EDOT and (–)-myrtenal with POCl_3_. The reaction was carried out in a Schlenk tube.

**Figure 1:**
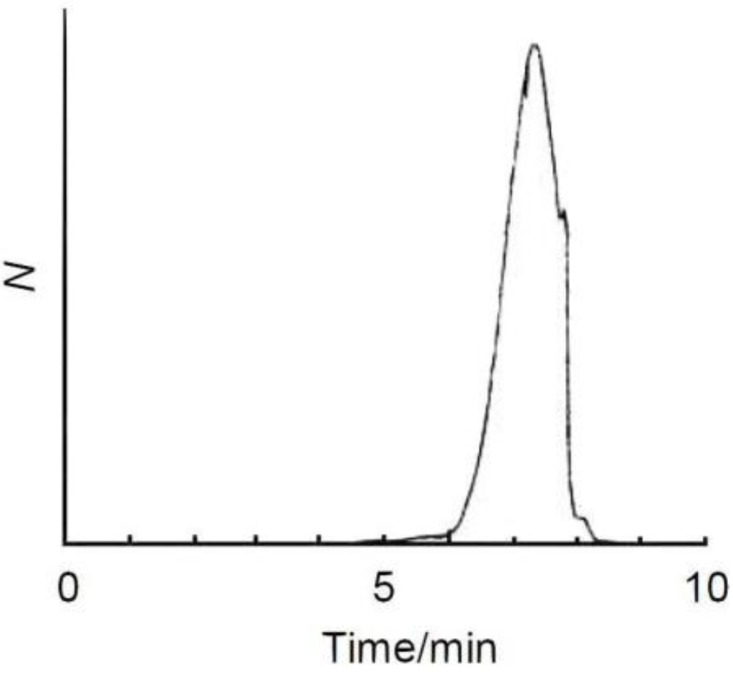
GPC curve of ME1.

A solution of EDOT (0.50 g, 3.5 mmol), (–)-myrtenal (0.54 g, 3.6 mmol), and POCl_3_ (0.061 g, 0.40 mmol) in 1,4-dioxane (5 mL) was refluxed at 80 °C for 25 h (molecular structure of EDOT and (–)-myrtenal are shown in Scheme 2). The reaction mixture was poured into a large amount of methanol. Then, aqueous ammonia was added to remove the catalyst from the resultant. The crude product was purified by several washes in methanol. The precipitate was filtered off and dried under reduced pressure to yield the desired compound as a brown color solid. ^1^H NMR (400 MHz, *δ* from TMS (ppm), CDCl_3_): *δ* 0.73–0.96, 1.23–1.42, 1.85–1.97, 2.17–2.64, 2.98, 4.13, 6.06–6.39, 6.73. GPC measurement evaluated that the number average molecular weight (*M*_n_) is 900, the weight average molecular weight (*M*_w_) is 1600, polydispersity (*M*_w_/*M*_n_) is 1.72. This result indicates that the resultant is an oligomer with molecular weight dispersion (Figure 1). Employment of EDOT as an arylene unit can be expected to avoid side reaction at β and β′ positions in the oxidative polycondensation because these parts are protected by the ethylenedioxy group.

## 3. Results and Discussion

**Figure 2:**
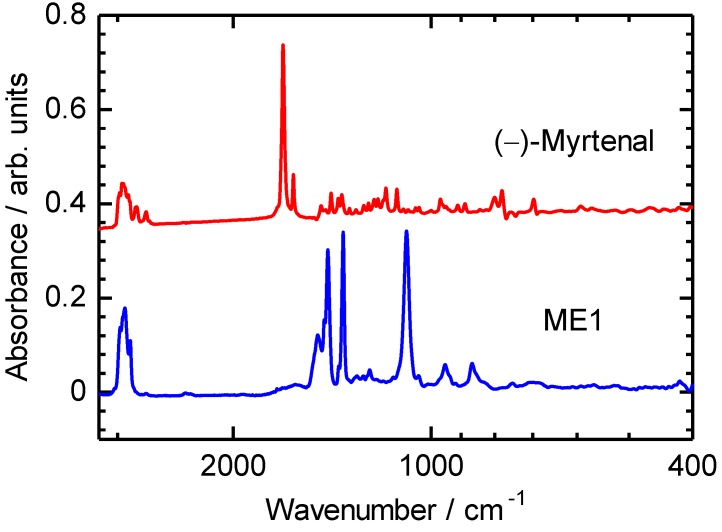
IR spectra of ME1 and (–)-myrtenal.

IR absorption spectra of (–)-myrtenal and ME1 are examined, as shown in Figure 2. An absorption band of C−H stretching vibration of the aldehyde group of (–)-myrtenal is observed at around 1700 cm^−1^. On the other hand, IR absorption spectrum of ME1 shows no signals at around 1700 cm^−1^. The absence of the aldehyde signal in the IR absorption spectrum of ME1 indicates completion of the polycondensation reaction [34,35]. Characteristic absorption bands at 1089 cm^−1^ and 1360–1490 cm^−1^ of ME1 are attributable to C–O–C asymmetric stretching vibration and C=C stretching vibration of EDOT, respectively. ^1^H NMR measurements of ME1 and (–)-myrtenal were also performed for verification of completion of the reaction. NMR signals of ME1 were totally broadened. Typical signal at 9–10 ppm attributed to aldehyde group disappears after the reaction. This can be due to the fact that the polycondensation between the aldehyde group and arylene units was successfully carried out. The characteristic signals of EDOT (4.16 ppm, –O–C*H*_2_–C*H*_2_–O–) and (–)-myrtenal (0.7–2.9 ppm, each alkyl group) were observed in ME1 spectrum except aldehyde signal [36]. This result agrees with the IR measurements.

**Figure 3:**
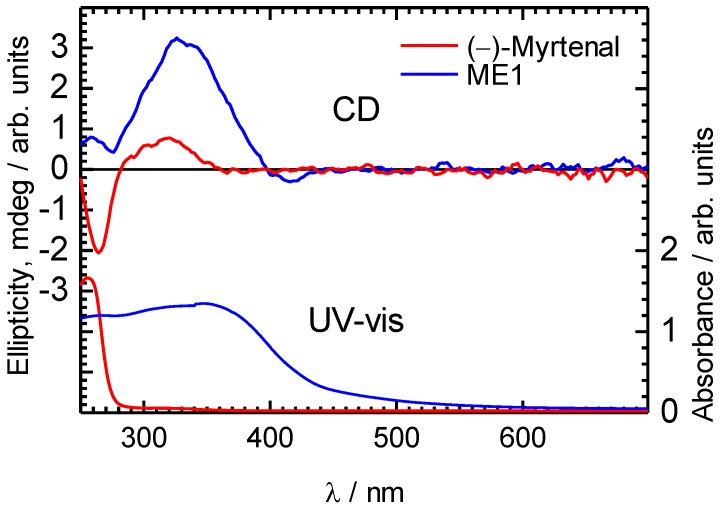
CD spectra of ME1 and (–)-myrtenal in CHCl_3_ solution.

UV-vis absorption measurements of (−)-myrtenal and ME1 in CHCl_3_ were examined (Figure 3). A broad absorption band of ME1 indicates that the π-conjugation length of the backbone is developed. An optical bandgap of ME1 calculated from the absorption spectrum is to be 2.8 eV. Therefore, ME1 is considered to be a semiconducting organic material. Although the previously reported methine type polymers exhibited extended π-conjugation and low-bandgap because of benzenoid and quinonoid alternating structure in the main chain, the bandgap value in the present study is relatively large [28,37]. This is mainly because of the low degree of the polycondensation. Besides, bulky (–)-myrtenal moieties in the side chain might decrease the coplanarity of the main chain.

**Figure 4:**
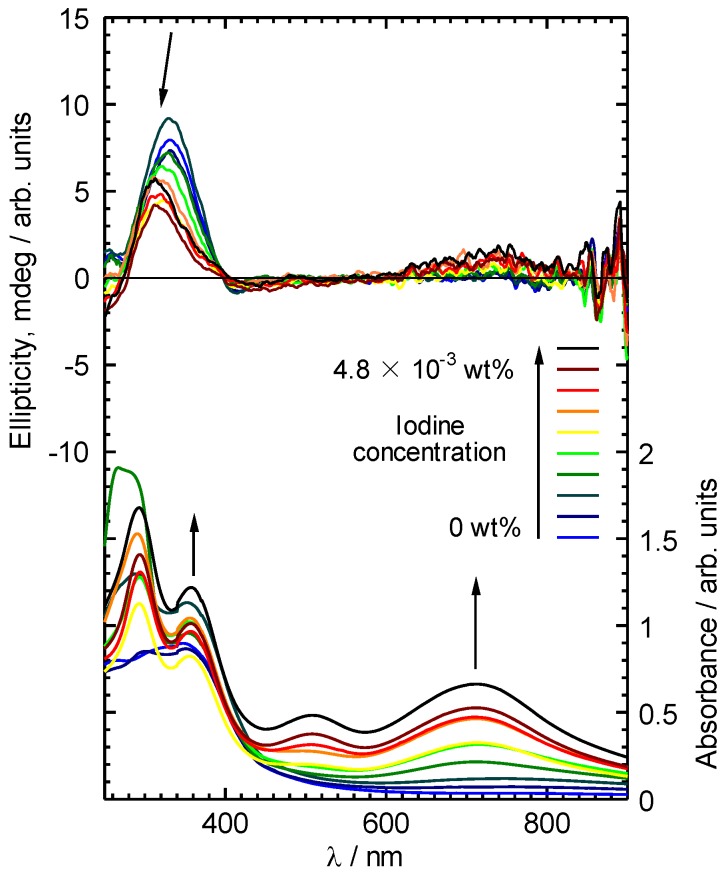
CD and UV-vis absorption spectra of ME1 in CHCl_3_ with various iodine doping levels.

**Figure 5:**
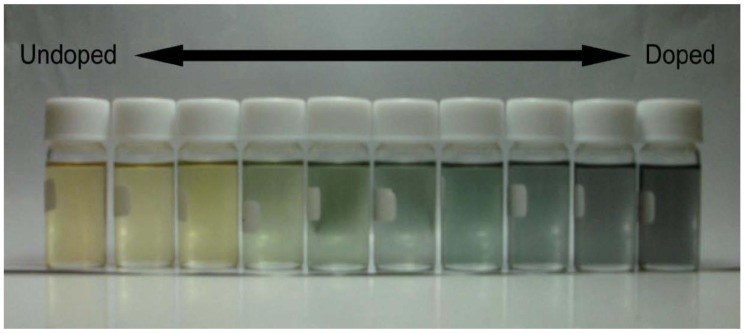
Color change of ME1 in CHCl_3_ solution from orange to green with iodine doping.

**Figure 6:**
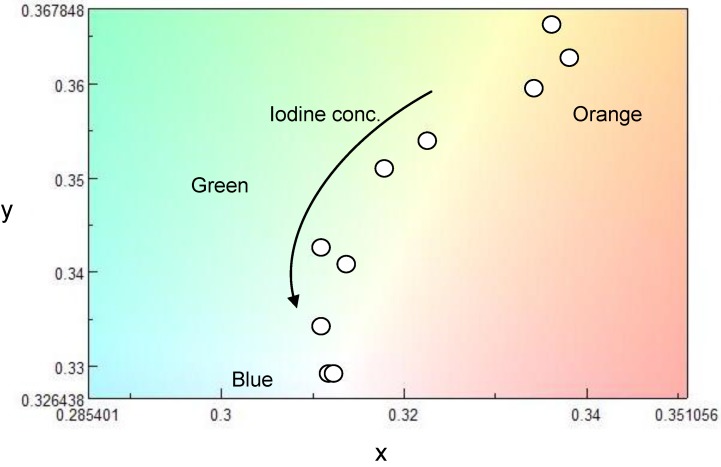
Change in CIE color space coordination diagram of ME1 with iodine doping.

CD absorption spectra of ME1 and (–)-myrtenal were obtained in CHCl_3_ solution, as shown in Figure 3. Both spectra show clear CD curves. Broad CD signal of ME1 indicates that the backbone of ME1 individually shows chiroptical activity. After the polycondensation, chiroptical activity of the side chain remains and it induces chiroptical activity for the entire system. Such oxidative polycondensation can be simple and convenient method to introduce chiroptical activity for conjugated materials. We examined a doping effect of iodine (electron acceptor) for ME1 with UV-vis optical absorption, CD, and ESR. Figure 4 shows CD and UV-vis absorption spectra of ME1 in CHCl_3_ with various iodine doping levels in solution. As for UV-vis spectra, clear changes in UV-vis absorption spectrum of ME1 were observed upon progress of the doping. New absorption peaks at 295 nm and 355 nm appeared with progress of the iodine doping. These two peaks indicate an occurrence of oxidation of ME1 by the doping. Furthermore, broad absorption band at 600−800 nm appeared gradually. This suggests generation of radical cations (charge species, polarons) for ME1 [38]. CD spectra of ME1 by the iodine doping show blue shift at short wavelengths and decrease of the intensities by the iodine doping. The blue shift implies that the short helical form induced by the chiral substituent is released by the doping. Color change of ME1 in solution with various iodine doping levels is shown in Figure 5. Original color of iodine in a solution is purple. However, addition of iodine leads changes in color for the material from orange to dark green. This result suggests that the iodine doping tunes the color and chiroptical activity of ME1. Commission Internationale de l’Eclairage (International Commission on Illumination, CIE) color space confirms the color in the solution, as shown in Figure 6.

**Figure 7:**
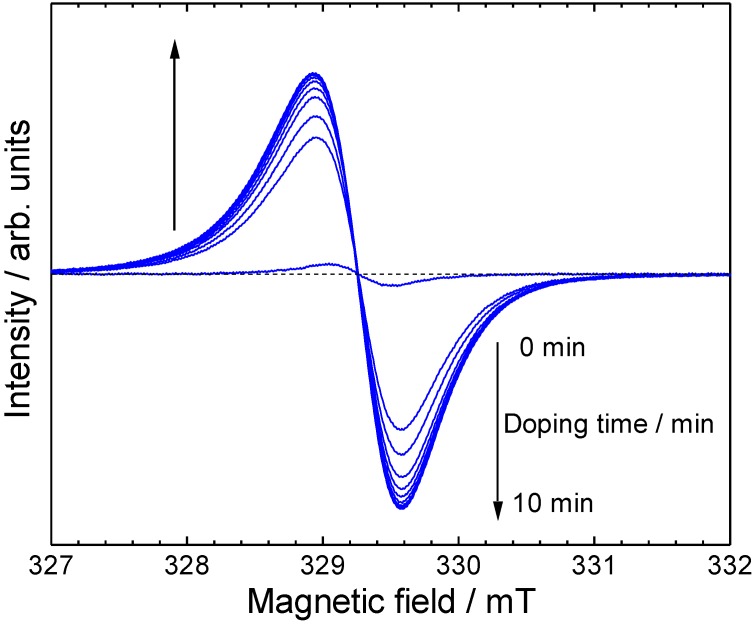
*In-situ* ESR spectra during vapor phase iodine doping.

**Figure 8:**
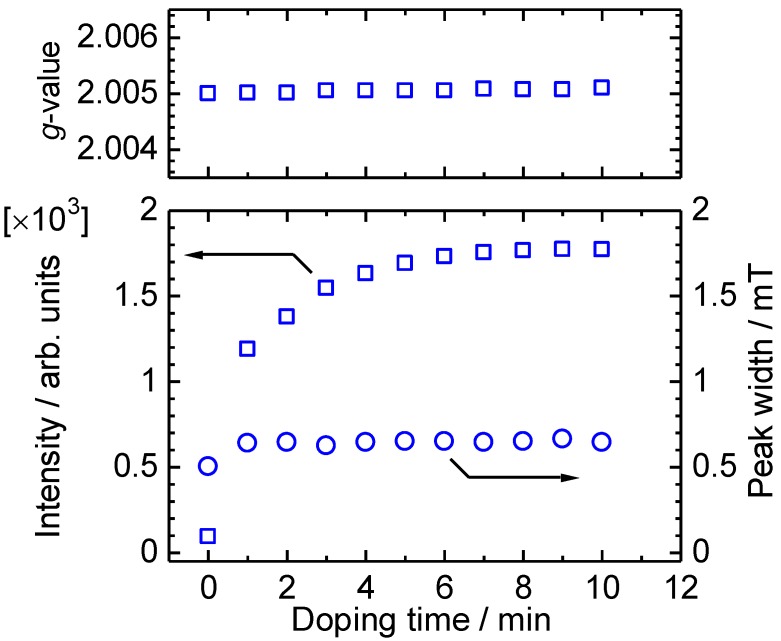
*g*-value, intensity, and peak width (Δ*H*_pp_) of ME1 with vapor phase iodine doping for 0–10 min.

*In-situ* ESR spectra of ME1 with vapor phase iodine doping were measured (Figure 7). As prepared oligomer shows weak ESR signal, indicating incomplete dedoping of POCl_3_ with aqueous ammonia treatment, or doping with oxygen in the air. The spin concentration increased rapidly upon the doping. This indicates a generation of radical cations along the main chain. Figure 8 shows *g*-value, intensity, and peak width (Δ*H*_pp_) plots *vs*. the doping time. The peak width and *g*-value are constant upon the doping. However, the intensity is increased by the doping, indicating that the doping is effectively carried out and charge species are generated along the main chain [39].

## 4. Conclusions

A new methine π-conjugated oligomer was synthesized from EDOT and (–)-myrtenal. This shows a broad absorption band and a CD signal in the long wavelengths. The chiral side chain induces helical conformation of the main chain to exhibit chiroptical activity. Iodine doping for the oligomer was examined, and the doped state was monitored with the UV-vis optical absorption, the CD, and the ESR spectroscopy. The UV-vis absorption and the ESR measurements confirm the formation of radical cations. CD spectra of the oligomer show blue shift of the CD signal during the doping. This suggests that the change in electronic structure upon doping tunes its chiroptical activity.
